# Biomimetic polyetheretherketone microcarriers with specific surface topography and self-secreted extracellular matrix for large-scale cell expansion

**DOI:** 10.1093/rb/rbz032

**Published:** 2019-09-30

**Authors:** Qingming Ji, Zongliang Wang, Zixue Jiao, Yu Wang, Zhenxu Wu, Peng Wang, Yuhang Zhu, Shuo Sun, Yi Liu, Peibiao Zhang

**Affiliations:** 1 Department of Spine Surgery, The First Hospital of Jilin University, Changchun 130021, PR China; 2 Key Laboratory of Polymer Ecomaterials, Changchun Institute of Applied Chemistry, Chinese Academy of Sciences, Changchun 130022, PR China; 3 University of Science and Technology of China, Hefei 230026, PR China; 4 Department of Orthopedics, China-Japan Union Hospital, Jilin University, Changchun 130033, PR China

**Keywords:** PEEK, topological microcarriers, recycle, decellularization, cell adhesion

## Abstract

Reusable microcarriers with appropriate surface topography, mechanical properties, as well as biological modification through decellularization facilitating repeated cell culture are crucial for tissue engineering applications. Herein, we report the preparation of topological polyetheretherketone (PEEK) microcarriers via gas-driven and solvent exchange method followed by hydrothermal treatment at high temperature and pressure. After hydrothermal treated for 8 h, the resulting topological PEEK microcarriers exhibit walnut-like surface topography and good sphericity as well as uniform size distribution of 350.24 ± 19.44 µm. And the average width between ravine-patterned surface of PEEK microcarriers is 780 ± 290 nm. After repeated steam sterilization by autoclaving for three times, topological PEEK microcarriers show nearly identical results compared with previous ones indicating strong tolerance to high temperature and pressure. This is a unique advantage for large-scale cell expansion and clinical applications. Moreover, PEEK microcarriers with special topography possess higher protein adsorption efficiency. In addition, the reutilization and biofunctionalization with repeated decellularization of topological PEEK microcarriers show highly beneficial for cell adhesion and proliferation. Therefore, our study is of great importance for new generation microcarriers with micro-and nano-scale surface feature for a broad application prospect in tissue engineering.

## Introduction

**Scheme 1:**
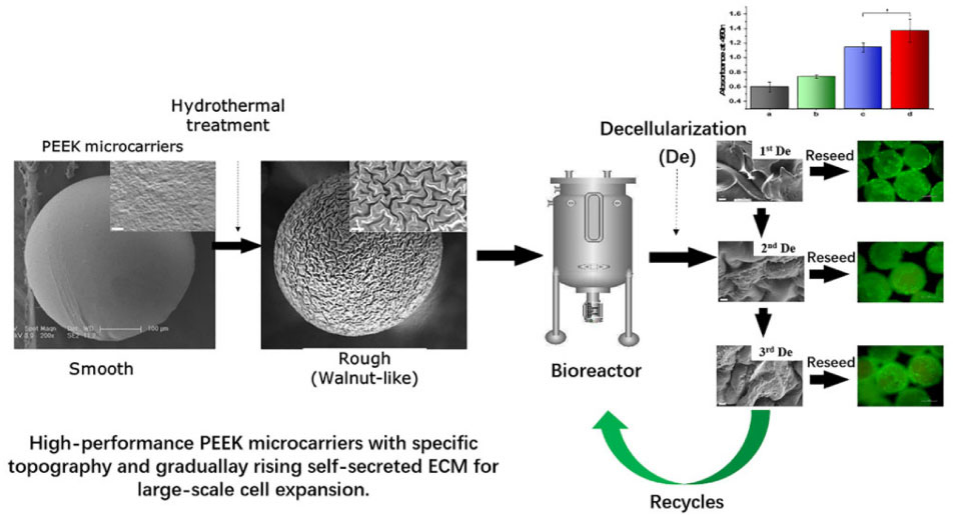
Schematic diagram of the process of fabricating topological PEEK microcarriers and promoting cell adhesion after decellularization.

In this study, we report the preparation and surface morphology improvement of polyetheretherketone (PEEK) microcarriers via gas-driven and solvent exchange method followed by hydrothermal treatment at high temperature and pressure (Scheme 1). The resulting topological PEEK microcarriers exhibit (i) walnut-like surface topography, (ii) repeatable steam sterilization by autoclaving, (iii) higher protein adsorption efficiency, (iv) better cell adhesion and proliferation and (v) reutilization and biofunctionalization by repeating decellularization.

Large-scale cell culture has become a focus of research for clinical cell therapies in applications and cell expansion in tissue engineering [1, 2]. Traditional planar cell culture systems have so many disadvantages such as limited specific surface or utilization rate of space, inhomogeneous diffusion of nutrients and metabolites and low scale-up capability affecting the final productivity that they are not suited for cell expansion at a large scale [3]. One of the choices for overcoming this obstacle would be a microcarrier-based culturing technology. In order to achieve desired cell numbers, microcarrier technology is expected to play a vital role in the successful introduction of novel and promising methods for cell expansion [4]. Microcarriers are spheres that can act as the support matrix for the growth of anchorage-dependent cells in large-scale cultivation. In 1967, van Wezel reported microcarrier for the first time, diethylaminoethyl-Sephadex A50. Since then, the commercialization of microcarriers including Cytodex-1, Cytodex-2, Cytodex-3, Plastic Plus Coated, Glass Coated and Hillex has been largely developed [5]. Microcarriers are widely used in various biomedical applications, including drug delivery, tissue engineering and cell therapy [1, 2, 4]. Compared with two dimensional (2D) conventional cell culture system, three dimensional (3D) microcarriers have been showing great advancement and can mimic *in vivo* cellular microenvironment where cell-cell interaction occurs in higher dimensions. Owing to the large specific area of microcarriers, which facilitates the sufficient exchange of nutrients, oxygen and metabolic waste within the culture medium, they are quite efficient for cell amplification [6]. Additionally, microcarriers have a 3D spatial structure similar to the environment of cells *in vivo* and they are beneficial for cell migration and proliferation resulting enhanced scale-up production capacity [7, 8].

Many efforts have been made to fabricate microcarriers with appropriate surface topography, reutilization, mechanical properties, as well as biological modification, such as decellularization [2, 4, 9]. The ideal microcarrier for adherent cell culture should be nontoxic, biocompatible and promote efficient cell adhesion and robust cell proliferation, and at the same time fulfill adequate physical and mechanical properties for dynamic systems [4, 10]. Surface topography plays an important role in a biological system for most biological reactions occurring at the surfaces and interfaces. Surface feature may influence the adsorption of proteins and the potential for cells to adhere and proliferate [11, 12]. The sterilization and reutilization of microcarriers are important factors affecting the cost-effectiveness of the production process [2]. The required number of microcarriers for large-scale industrial amplification is huge, while the reutilization rate of microcarriers is not high. Therefore, the design and fabrication of reusable microcarriers play an important role in the biomedical applications [13]. There is an urgent need for a new commercially available microcarrier that can be reused for cultivation of anchorage dependent cells. In addition to the heat tolerance for steam sterilization, the microcarrier should be rigid enough in order to support cell spreading and withstand the shear forces encountered during the 3D cell cultivation.

Polyetheretherketone (PEEK) is a special function thermoplastic which has been widely used for biomedical applications with excellent biocompatibility and chemical resistance, high mechanical properties and elasticity modulus similar to human bone [14, 15]. In this study, it was the first time to prepare PEEK microcarriers with topological morphology using gas-driven and solvent exchange method followed by hydrothermal treatment as a new platform for cell expansion. The purpose of this study is to overcome the limitations of bioinert feature and smooth surface of PEEK microcarriers which are not conducive to cell attachment. To alter the surface topography and chemistry of PEEK materials, much work has been conducted and it can be classified into two general approaches: coating deposition technique and direct surface modification technique [16, 17]. The surface roughness of implants has been suggested to be important for cell attachment. Various approaches such as mechanical texturing, chemical etching, plasma treatment, sand blasting and laser patterning have been used to modify the degree of surface roughness as well as topography of PEEK [17]. Several investigations into PEEK materials have begun to support that a moderated degree of roughness exhibited significant better cell attachment efficiency [11, 18]. Some studies also support that surface topography plays a larger role than chemical composition, with micro-and nano-scale features exhibiting improved functional ability of cell attachment compared with smooth surfaces [18–20]. Cell-surface interactions play a crucial role for applications of microcarriers in tissue engineering. Therefore, it is evident that not only the chemical composition [21, 22], but also the surface topography of substrates affect cell adhesion and proliferation [23, 24].

## Results and discussion

**Figure 1:**
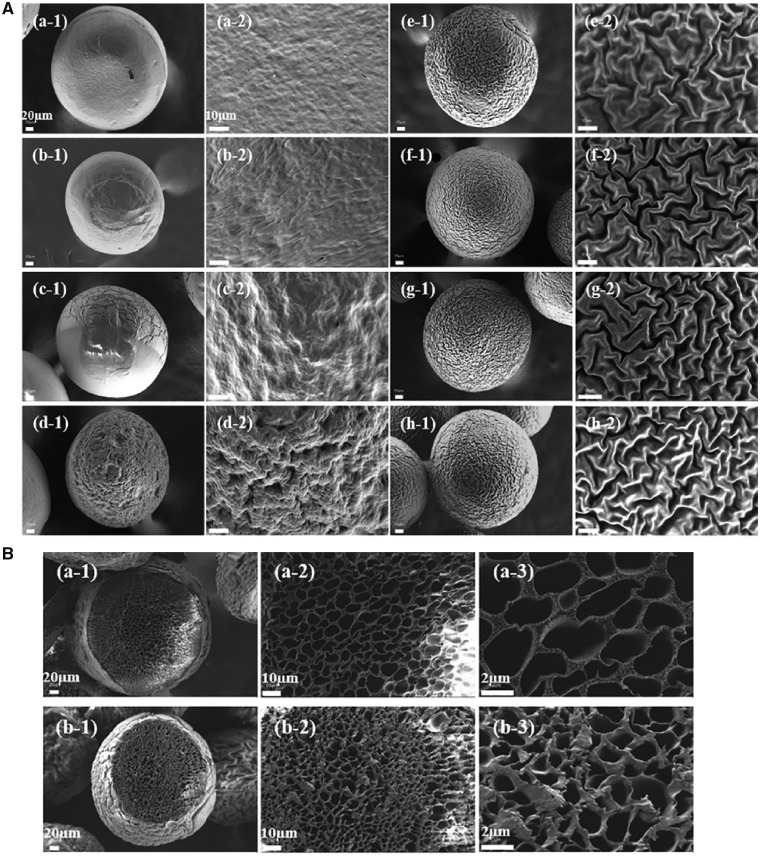

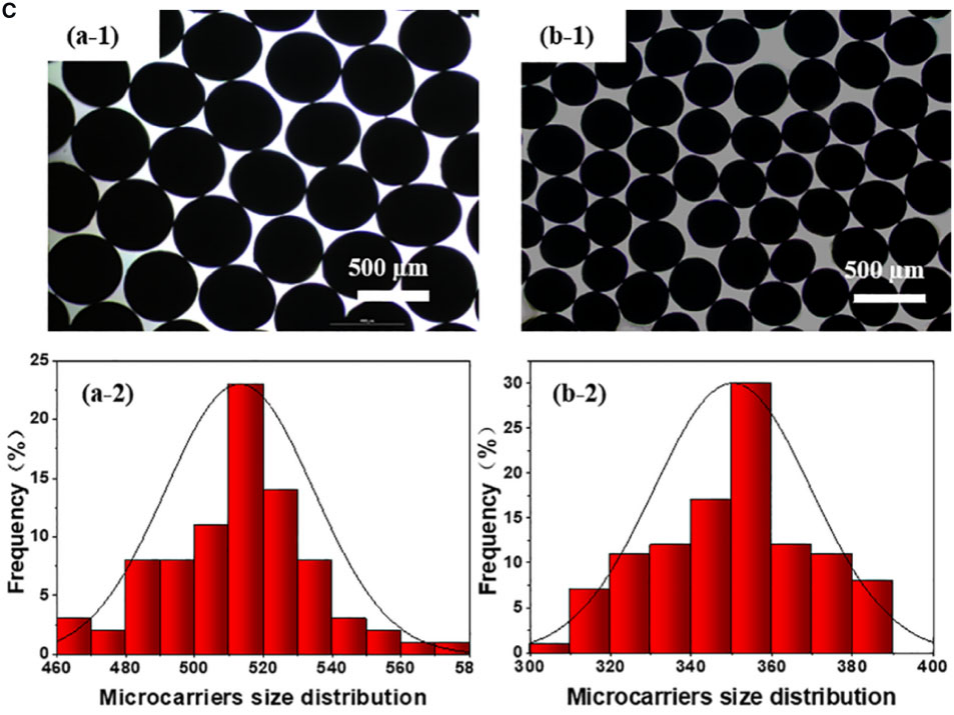
(**a**) SEM images of surface morphological features of PEEK microcarriers before (a) and after hydrothermal treatment at 180°C for 0.5 (b), 1 (c), 2 (d), 4 (e), 8 (f), 12 (g) and 24 h (h). All the scale bar lengths for low (-1) and high (-2) magnification are 20 and 10 μm, respectively. (**b**) SEM images of cross-section morphological features of PEEK microcarriers before (a) and after hydrothermal treatment at 180°C for 8 h (b). All the scale bar lengths for (-1), (-2) and (-3) are 20, 10 and 2 μm, respectively. (**c**) The micrographs (-1) and size distribution (-2) of PEEK microcarriers before (a) and after hydrothermal treatment at 180°C for 8 h (b). All the scale bar lengths are 500 μm

In the present study, we fabricated PEEK microcarries with special topography via hydrothermal treatment at high temperature and pressure. This method was simple and feasible to form roughened and wrinkled surface topography for enhancing protein adsorption capacity, further improving osteoblasts attachment and proliferation. PEEK powder was dissolved in concentrated sulfuric acid and the homogenous solution was dripped at a constant flow rate through a tube assisted by nitrogen flow and PEEK microcarriers with smooth surface were obtained. Then the smooth PEEK microcarriers were treated with deionized water in a reactor at 180°C for 0.5, 1, 2, 4, 8, 12 and 24 h, respectively. As shown in Fig. 1a, scanning electron microscope (SEM) images demonstrated that PEEK microcarriers became a little smaller and wrinkled structures gradually appeared on the surface of microcarriers. As hydrothermal treated for 0.5–8 h, the ravine structure became more apparent. The surface topography of the PEEK microcarriers looked like a walnut and it was quite regular. After hydrothermal treatment for 4, 8, 12 and 24 h, the average width between ravine-patterned surface of PEEK microcarriers was 0.6 ± 0.22, 0.78 ± 0.29, 0.79 ± 0.24 and 0.74 ± 0.25 µm, respectively. Surface modification such as surface topography, surface roughness, micro-and nano-scale surface feature, are often needed to enhance the interactions between cells and the materials [25]. However, after hydrothermal treatment of 12 and 24 h, the change of surface morphology and size was not obvious compared with that of 8 h. Therefore, considering of energy conservation, processing for 8 h was chosen as the optimum time of hydrothermal treatment for the following experiments and the resulting microcarriers were named as topological PEEK microcarriers. In addition, honeycomb-like pore structure was observed for cross-section of topological PEEK microcarriers which was similar to cancellous bone structure, and the pore structure was more dense after hydrothermal treatment for 8 h (Fig. 1b). Light microscope observation showed that PEEK microcarries exhibited good sphericity and uniform size distribution of 513.11 ± 21.61 µm before heating and 350.24 ± 19.44 µm after heating for 8 h, respectively (Fig. 1c). It has been reported that wrinkling referred to regular or irregular undulations appearing on an initially flat film surface, which occurred when there was an anisotropic or inhomogeneous volumetric compression resulting from structural heterogeneities, boundary constraints and/or non-uniform microenvironment [26, 27]. A possible mechanism of the wrinkled-surface morphology formation in this study could be proposed as follows. The glass transition temperature (*T*_g_) of PEEK is 143°C [15]. When the temperature of hydrothermal treatment was up to 180°C (> 143°C), the smooth surface of PEEK microcarriers shrinked uniformly leading to the formation of wrinkled surface, and increased the surface roughness with different heating time. The rigidity of PEEK had an effect of supporting or impeding in the process of polymer shrinkage, which was helpful to the appearance of ravine structure. As the processing time increased up to 8 h, PEEK microcarriers thus appeared tunable ravine morphology with wrinkled-surface. In later stages (12 and 24 h), there was no significant change for the degree of wrinkling and ravine morphology and it might be attributed to higher critical strain needed to generate wrinkling with increasing shrinkage degree and mechanical stiffness [28–30]. Compared with previously reported techniques for increasing surface roughness [17, 30, 31], our modification method is simple and efficient to prepare microcarriers with controlled surface morphology requiring less technical limitations.

**Figure 2:**
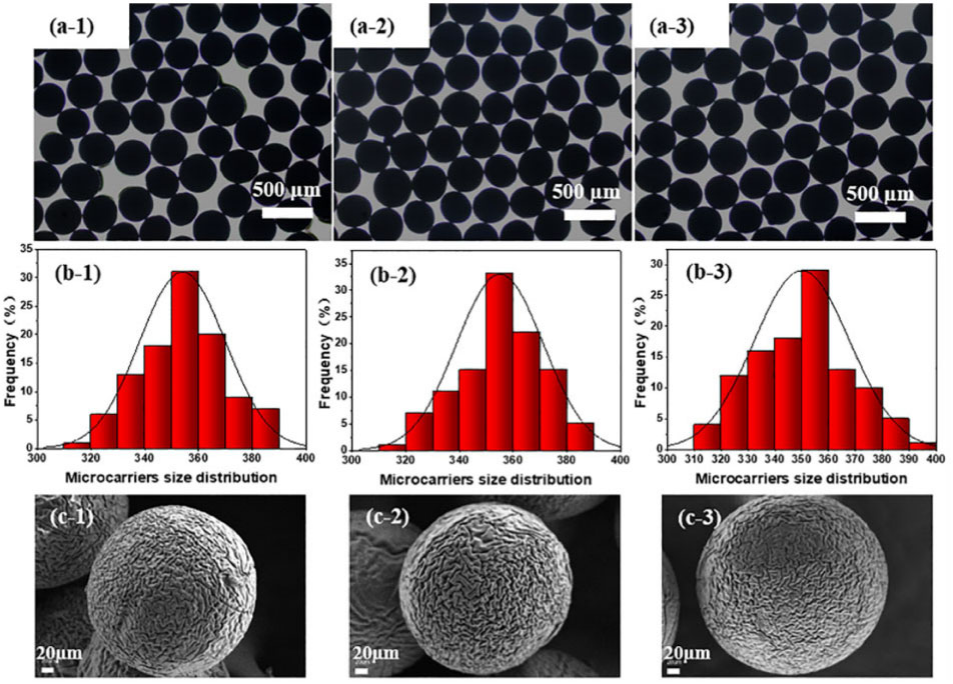
Steam sterilization resistance test of PEEK microcarriers which have been hydrothermal treated at 180°C for 8 h. The micrographs (**a**), size distribution (**b**) and SEM images (**c**) of repeated sterilized microcarriers in an autoclave for 1 time (-1), 2 times (-2) and 3 times (-3). The scale bar lengths for a-1 to a-3 and c-1 to c-3 are 500 and 20 μm, respectively

To be universally known, sterilization is an important and indispensable process for microcarriers in cell expansion. High-pressure steam sterilization is a simple, convenient and low-cost sterilization method. So the reutilization of topological PEEK microcarriers prepared in this study was tested three times by autoclaving under 121°C for 20 min. Light microscope observation showed that PEEK microcarries exhibited good sphericity and uniform size distribution of 353.96 ± 15.88, 355.03 ± 16.04 and 350.00 ± 17.72 µm after steam sterilization for 1, 2 and 3 times, respectively (Fig. 2). The SEM images of PEEK microcarriers after steam sterilization for 1–3 times showed that nearly identical surface topography was obtained compared with previous ones (Fig. 2). The results implied that repeated steam sterilization did not affect the size, sphericity and morphology of the topological PEEK microcarriers indicating strong tolerance to high temperature and pressure. This is a unique advantage for large-scale cell expansion and clinical applications.

**Figure 3:**
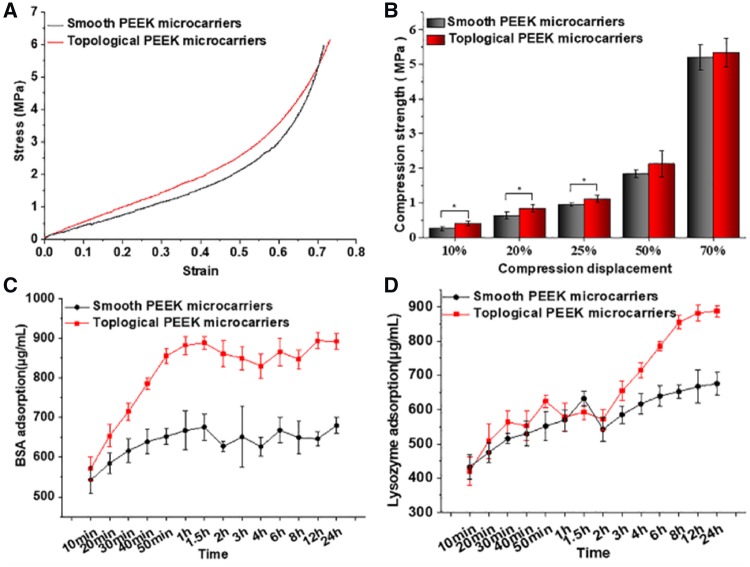
(**a**) Stress-strain curve and (**b**) compressive property of the molded PEEK microcarriers. *P <* *0.05. (**c**) Time-dependent BSA and (**d**) lysozyme adsorption capacity of different samples from 10 min to 24 h. The concentration of BSA and lysozyme solution: 1 mg/ml, pH = 7.35, temperature: 37°C

To evaluate the mechanical properties of the obtained microcarriers, a mold was designed and shown in Supplemental Fig. S2. The compression strength of accumulated topological PEEK microcarriers was higher than that of smooth group at different displacements, especially at 10, 20 and 25% displacements (*P *<* *0.05, Fig. 3a and b). As the stress increased, both groups were eventually flattened and rammed. This phenomenon was presumably due to the surface of topological PEEK microcarries shrinked uniformly and they were more dense than the smooth ones resulting better mechanical properties. When put in bioreactors, topological PEEK microcarriers can provide efficient support and stiffness to withstand the shear stress in stirring culture systems.

The surface features of microcarriers can significantly influence the adsorption of proteins. The first step of exposure to a biological environment for any microcarriers would be rapid adsorption of proteins to its surface [32]. Then cell attachment might occur rapidly on the protein-coated surface [33]. So the immobilization of proteins, enzymes or peptides on microcarriers is a major aim of biochemical surface modification. In our study, bovine serum albumin (BSA) and lysozyme were selected as model proteins to investigate the protein adsorption ability for the prepared PEEK microcarriers. That is because the two proteins possess different isoelectric point of 4.7 and 10.8, respectively. As shown in Fig. 3c and d, BSA adsorption was up to 893.46 µg/ml for the topological samples compared with that 646.96 µg/ml for smooth samples. Lysozyme adsorption was 881.71 µg/ml for the topological samples, while it was only 667.58 µg/ml for that of smooth microcarriers. The results implied that the protein absorption capacity of topological PEEK microcarriers was obviously enhanced due to the increased surface roughness compared to smooth ones. Cells in their surrounding environment are anchored by attachment to the proteins. The cell surface has a large family of receptors that interact with proteins which plays a crucial role in stimulating a series of cell response [34]. Therefore the increased adsorption of proteins is beneficial for cells to adhere and proliferate on the microcarriers.

**Figure 4:**
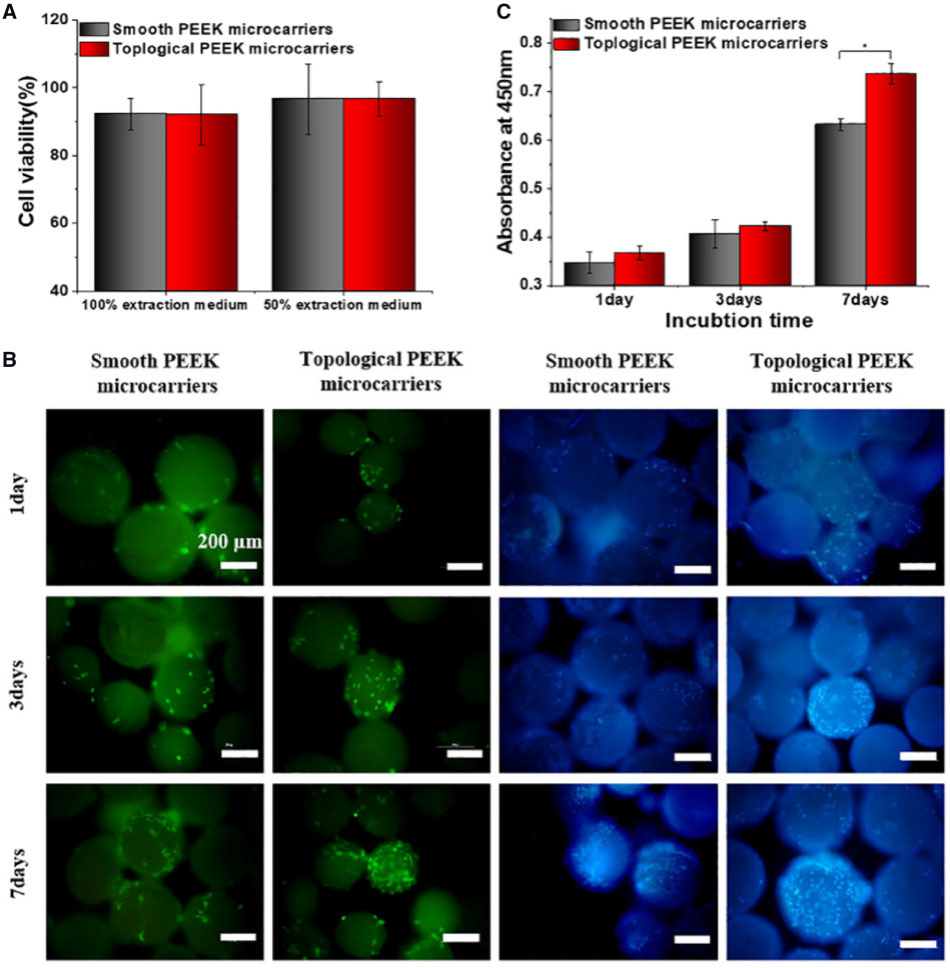
(**a**) *In vitro* cytotoxicity of different microcarriers evaluated by 24 h indirect extraction liquids assay. (**b**) Micrographs of adherent cells on the smooth and topological microcarriers incubated for 1, 3 and 7 days. The cells were stained with calcein AM (green) and DAPI (blue, nuclei). All the scale bars were 200 μm. (**c**) Cell proliferation of MC3T3-E1 cultured on the different PEEK microcarriers for 1, 3 and 7 days was evaluated by CCK-8 assay, *P <* *0.05

For cell experiments, we first investigated the cytotoxicity through examining the viability of MC3T3-E1 cells. The cytotoxicity tests were carried out by indirect contact. Fig. 4a showed the cell viability cultured in 100 and 50% extraction medium for 24 h, respectively. The cell viability was 92.19 and 92.09% for smooth and topological samples in 100% extraction medium (*P *>* *0.05). When the extraction was diluted for 1-fold, the cell viability was 96.59 and 96.60% for smooth and topological samples, respectively (*P *>* *0.05). It could be seen that cell viability in the two groups of extracts showed no significant difference. Despite the PEEK powder was dissolved in concentrated sulfuric acid in the fabrication process, the original extracts and dilution for 1-fold did not exhibit acute cytotoxicity. That was because the samples were immersed in deionized water and subsequent hydrothermal treatment could decrease sulfur content attenuating cytotoxicity [35]. So indirect cytotoxicity assays indicated that the obtained PEEK microcarriers were nontoxic and safe for application *in vivo*.

In addition, surface properties may also affect the potential for cell adhesion and proliferation. In this study, MC3T3-E1 cells were fluorescently stained to assess cell adhesion on the microcarriers. As shown in Fig. 4b, cell attachment on the topological samples was better than that of smooth ones at 1, 3 and 7 days. The adherent cell numbers on topological PEEK microcarries were more than that of smooth PEEK samples. Especially for 7 days, microcarriers were almost completely covered by cells. It would be attributed to the uniform regular wrinkled surface morphology was more compatible for cell attachment. Increased optical density (OD) values in Fig. 4c after culturing for 1, 3 and 7 days showed MC3T3-E1 cell proliferation on the different samples. Especially, after culturing for 7 days, topological PEEK microcarriers exhibited significant higher OD values than smooth ones (*P *<* *0.05). The results showed that cell proliferation on the topological PEEK microcarriers was significantly enhanced and the topographically different surfaces with the same chemical properties could lead to diverse cellular response which was similar with previous studies [19, 36]. Therefore, the surface roughness and topography may influence the cellular response of osteoblasts to PEEK microcarriers and cell adhesion and proliferation was improved by the micro-and nano-scale feature compared with the smooth surfaces.

**Figure 5:**
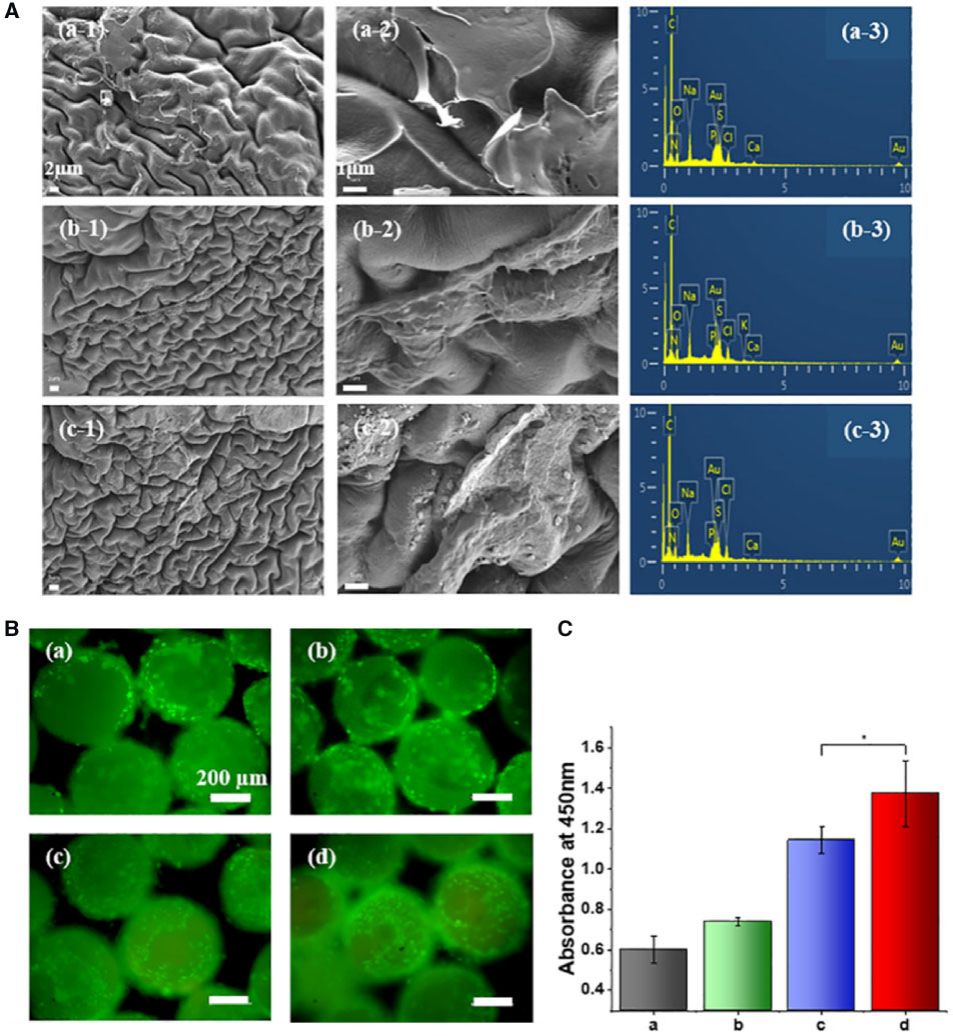
(**a**) SEM micrographs (-1 and -2) and EDX analysis (-3) of the repeated decellularized PEEK microcarriers for 1 time (a), 2 times (b) and 3 times (c). All the scale bars for (-1) and (-2) were 2 and 1 μm. (**b**) Micrographs of adherent cells on the original topological (a), decellularized for 1 time (b), 2 times (c) and 3 times (d) of PEEK microcarriers incubated for 7 days, respectively. The cells were stained with calcein AM (green). All the scale bars were 200 μm. (**c**) The quantification of cell proliferation evaluated by CCK-8 assay, *P <* *0.05

Although microcarriers made from PEEK showed good reproducibility and mechanical properties, lacked cell recognition sites might limit cell adhesion and growth [11]. Furthermore, the characteristics of coating on materials are important factors influencing cell adhesion and proliferation on the microcarriers [2, 37]. In order to further improve the performance of cell adhesion and proliferation, the topological PEEK microcarriers were specially treated and extracellular matrix (ECM) was deposited on the surface of microcarriers using repeated decellularization strategy. MC3T3-E1 cells were seeded on the sterilized microcarriers and cultured for 7 days, decellularization process was performed using 0.5 wt% of sodium dodecyl sulfate (SDS) to produce cell-derived ECM [38]. The obtained ECM-encapsulated PEEK microcarriers were sterilized again and reseeded with MC3T3-E1 cells. After culturing for another 7 days, decellularization process was performed again. Then the sterilization, cell seeding, culturing and decellularization process was repeated again. After decellularization for three times, observation from SEM (Fig. 5a) showed components of ECM have been coated to the surface of PEEK microcarriers. As decellularized for one time (Fig. 5a; a-1, a-2), PEEK microcarriers were covered with ECM secreted from cells and cell-derived ECM was stronger and denser on the PEEK microcarriers decellularized for three times (Fig. 5a; c-1, c-2). ECM proteins on the three groups remained the same as shown in the EDX data with the presence of nitrogen, carbon and oxygen elements (Fig. 5a; a-3, b-3 and c-3). The result suggested the presence of ECM proteins on PEEK microcarriers. Functional biological ECM consists mainly of collagen, proteoglycans, elastin and other specialized protein macromolecules that are synthesized and secreted by animal cells. These compounds are located on the cell surfaces or between the cells [39]. The encapsulated layer of ECM can provide a naturally occurring, complex set of physiologically functional signals for cell growth mimicking an *in vivo* microenvironment. As previously reported, polystyrene microcarriers coating with a commercially available xeno-free substrate were able to provide the necessary amount of ECM proteins required for the adhesion and proliferation of the cells on the microcarriers [9]. Carmelo and colleagues reported ready-to-use ECM-coated microcarriers were successfully implemented, improving system scalability and cost-effectiveness, as well as facilitating translation to good manufacturing practice [40]. Several studies have also shown that there was superiority for the application of cell-derived ECM as a scaffold [41, 42]. Cell-derived ECM can be created *in vitro* using osteoblasts and can be used as new cell culture substrate after decellularization treatment [43].

To evaluate the ability of decellularized PEEK microcarriers to support *in vitro* cell growth, MC3T3-E1 cells were seeded on the surface of PEEK microcarriers which were decellularized for different times and cultured for 7 days. As shown in Fig. 5b, cell attachment on the ECM encapsulated PEEK samples was better than that of original bare topological ones at 7 days. The adherent cell numbers on ECM encapsulated PEEK microcarries were more than that of original bare topological PEEK samples. Especially for decellularized for three times, the ECM encapsulated PEEK microcarriers were almost completely covered by cells which were uniformly distributed on the microcarriers. These results demonstrated that decellularized ECM deposition on the PEEK microcarriers enhanced cell biocompatibility. After 7 days of incubation, PEEK microcarriers decellularized for three times exhibited significant higher OD values than the others (*P *<* *0.05). The results (Fig. 5c) indicated that better cell proliferation efficiency was obtained with the ECM encapsulated microcarriers. Modification with ECM layer which provides a natural microenvironment can compensate for the bioinert of PEEK. The results demonstrated that functionalization of PEEK microcarriers using cell-derived ECM could be useful for improving the bioactivity of materials and providing suitable microenvironment for cell expansion.

Cell-microcarrier interactions play a crucial role for cell expansion and function in tissue engineering. It is evident that not only the chemical composition [21, 22], but also the surface topography of substrates affect cell adhesion and proliferation [23, 24]. Surface modification such as surface topography, surface roughness, micro-and nano-scale surface feature, is often needed to enhance the interactions between cells and microcarriers and the surface modification of microcarriers is extensively recognized as a key strategy in the design of the next generation of microcarriers [4]. In this study, surface topological PEEK microcarriers were prepared by hydrothermal treatment. The walnut-like special roughness improved protein adsorption, cell adhesion and proliferation. The microcarriers can be resterilized by high pressure steam not affecting their size and topography. This makes it possible for repeated decellularization which is highly beneficial for cell attachment and growth. It has great application prospects for large-scale cell expansion and bone filling *in vivo*.

## Conclusions

As a robust and scalable platform for culturing of adherent cells, great achievement has been obtained for microcarriers as cell expansion and delivery systems. In this study, we developed a reliable technique to fabricate PEEK microcarriers with controllable special topography via gas-driven and solvent exchange method followed by hydrothermal treatment for enhancing protein adsorption capacity as well as improving osteoblasts adhesion and proliferation. The topological PEEK microcarriers could be sterilized by autoclaving repeatedly and the microcarriers encapsulated with ECM through decellularization for different times could improve the adhesion and proliferation of MC3T3-E1 cells. So the PEEK microcarriers could not only support large-scale manufacture of adherent cells but also be reusable. It is expected that the PEEK microcarriers with special topography and decellularized matrix might show excellent recellularization ability *in vitro* and have positive effect on cellular expansion. Therefore our study is of great importance for developing new microcarriers with micro-and nano-scale surface feature and improving surface properties in tissue engineering. In the future, the special topological PEEK microcarriers could also be co-cultured with cells in bioreactor to form a tissue or organ. These microtissues would be the basis of regenerative medicine for improving health of human being. Moreover, microcarriers prepared in this study could be combined with biodegradable microcarriers *in vivo* to explore optimal composition ratios for bone repair and further researches have been carrying out to evaluate bone repair effect of topological PEEK microspheres *in vivo*.

## Funding

This research was financially supported by the National Natural Science Foundation of China (Projects. 51473164 and 51673186), the joint funded program of Chinese Academy of Sciences and Japan Society for the Promotion of Science (GJHZ1519), and the Special Fund for Industrialization of Science and Technology Cooperation between Jilin Province and Chinese Academy of Sciences (2017SYHZ0021).

*Conflict of interest statement*. None declared.

## Supplementary Material

rbz032-Supplementary_data
