# Supplementary material for: Biomimetic polyetheretherketone microcarriers with specific surface topography and self-secreted extracellular matrix for large-scale cell expansion
Source: Regen Biomater. 2019 Sep 30;7(1):109–18. doi: 10.1093/rb/rbz032 (PMC7233611; doi:10.1093/rb/rbz032)
Supplement: rbz032-Supplementary_data [file rbz032-supplementary_data.zip › rbz032-suppl_data/communication__Supporting__Information2019-08-20.docx]

Supporting Information

**Biomimetic polyetheretherketone microcarriers with specific surface topography and self-secreted** [**extracellular**](javascript:;) [**matrix**](javascript:;) **for large-scale cell expansion**

Qingming Ji ^a,b^, Zongliang Wang ^b^, Zixue Jiao ^b^, Yu Wang ^b^, Zhenxu Wu ^b^, Peng Wang ^b,c^,

Yuhang Zhu ^d^, Shuo Sun ^a,b^, Yi Liu *^a^ and Peibiao Zhang *^b^

^a^ Department of Spine Surgery, The First Hospital of Jilin University, Changchun 130021, PR China

^b^ Key Laboratory of Polymer Ecomaterials, Changchun Institute of Applied Chemistry, Chinese Academy of Sciences, Changchun 130022, PR China

^c^ University of Science and Technology of China, Hefei 230026, PR China

^d^ Department of Orthopedics, China-Japan Union Hospital, Jilin University, Changchun 130033, PR China

* corresponding authors.

E-mail addresses: [liuyi2015310@163.com](mailto:liuyi2015310@163.com), [zhangpb@ciac.ac.cn](mailto:zhangpb@ciac.ac.cn).

**Materials**

Polyetheretherketone (PEEK) powder was purchased from (VICTREX , England). Alcohol(≥99.7%) and concentrated sulfuric acid (95%-98%) were purchased from (Beijing Chemical Works, China) without performing any further purification. Deionized water was used during the whole experiment.

**Preparation of PEEK microcarriers**

The PEEK microcarriers preparation device (**Figure S1**) comprised three parts that included air pump, Nitrogen cylinder, Teflon syringe and needle. The objective of the air pump was to provide a stable airflow force while fabricating the microcarriers. The needle could be inserted into the three-way cock and the Nitrogen airflow controlled the dripping speed.


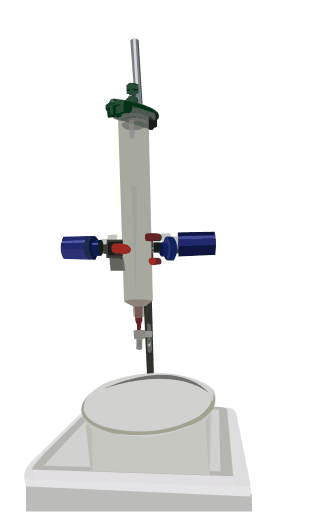

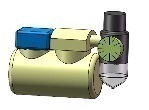

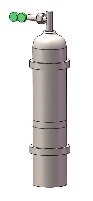

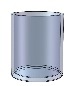


**Pump**

**Gas**

**Figure S1** The preparation device of PEEK microcarriers.

The whole preparation process of PEEK microcarriers was shown in Scheme 1, which involved gas-driven and solvent exchange method followed by hydrothermal treatment. The PEEK powder (1.8 g) was dissolved in concentrated sulfuric acid (30 mL) by mechanical stirring and then the homogenous solution was dripped into 30% ethanol aqueous solution (1000 mL) as the substitutional solution at a constant Nitrogen airflow rate (8 L/min) through a Teflon needle (27 G) to fabricate PEEK microcarries with smooth surface. The distance between the needle and the substitutional solution was set to be 14 cm. The microcarriers were immersed in deionized water for 48 h, and the water was changed every 8 h. Then the smooth PEEK microcarriers were hydrothermal treated with deionized water in the reactor (100 mL) at 180 °C for different times to obtain PEEK microcarriers with special topography.

**Surface morphology and chemical characterization**

A scanning electron microscope (SEM, Zeiss, Merlin, Gemini 2, Germany) was used to observe the surface morphology and cross-section morphological features of PEEK microcarriers before and after hydrothermal treatment. Energy dispersive X-ray spectrometry (EDX) (XL-30W/TMP, Philips, Japan) was employed to analyze the elemental composition after decellularization. In Figure 1, we measured the ravine from one side to the other side using Image J software. At least five widths including the widest and narrowest distances were selected for each ravine. The average width between ravine-patterned surface of PEEK microcarriers was shown in Table S1.

Table S1 The average width between ravine-patterned surface of PEEK microcarriers after different hydrothermal treatment time.

| Samples | Width between ravine-patterned surface (nm) |
| --- | --- |
| Hydrothermal treatment 4 h | 600±220 |
| Hydrothermal treatment 8 h | 780±290 |
| Hydrothermal treatment 12 h | 790±240 |
| Hydrothermal treatment 24 h | 740±250 |

**Measurement of microcarriers sizes**

The microcarriers before and after hydrothermal treatment were photographed using a phase contrast microscope (Nikon, TE-2000U). The diameter of the microcarriers was measured from their photomicrographs using Image J software.

**Repeated steam sterilization resistance test**

The obtained topological PEEK microcarriers were tested 3 times by autoclaving under 121 °C for 20 min. The micrograph, size distribution, surface morphology of microcarriers were characterized by microscope (Nikon, TE-2000U), Image J software and scanning electron microscope (SEM, Zeiss, Merlin, Gemini 2, Germany).

**Mechanical properties**

To evaluate the mechanical properties, we prepared a mold to measure the [compression](javascript:;) [strength](javascript:;) of accumulated PEEK microcarriers (**Figure S2**). Dry PEEK microcarriers with a diameter of 5 mm and a height of 35 mm in tube were accumulated for mechanical strength tests measured by the universal mechanical testing machine (Instron 1121, UK). The compressive strength was measured at a crosshead speed of 2 mm/min.

**
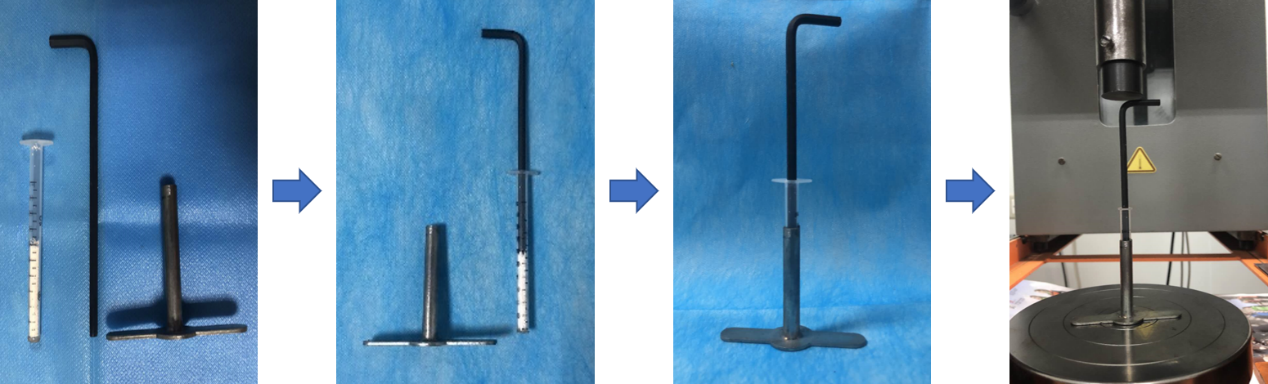
**

**Figure S2** The process to measure the [compression](javascript:;) [strength](javascript:;) of accumulated PEEK microcarriers.

**Protein adsorption**

The assessment of protein adsorption capacity onto the samples was performed according to a previous protocol.[[1](#_ENREF_1)] Bovine serum albumin (BSA) and lysozyme (Solarbio, Beijing, China) were selected as model proteins to investigate the protein adsorption ability for the PEEK microcarriers. That was because the two proteins possess different isoelectric point of 4.7 and 10.8, respectively. The specimens were immersed in 1mL BSA solution (pH = 7.35, 1 mg/mL) in each centrifuge tube under stirring at a constant rate of 150 rpm at 37 °C. The protein adsorption from 10 min to 24 h were evaluated. The adsorbed protein concentration was determined through the decrease of the concentration of BSA within the samples using BCA kit. The protein adsorption of lysozyme was performed using the same method.

**Cytotoxicity study**

Cytotoxicity of the different samples was evaluated by culturing MC3T3-E1 cells in extraction liquids. Briefly, the samples (100 mg) were immersed in 1 mL culture medium extracting for 24 h at 37 °C. MC3T3-E1 cells were seeded into 96-well plates at a density of 8 × 10^3^ cells per well. After 24 h of incubation, the media were replaced with 200 µL/well of the original extraction liquids and extraction liquids diluted for 1 fold, respectively. After 24 h, 20 µL CCK-8 was added into each well, and the incubation was kept for another 2 h and optical density was determined at 450 nm (Tecan Infinite M200). The complete cell culture medium were used as control groups. The cytotoxicity of the samples was expressed as cell viability ratio, which was calculated by the following equation: Cell viability (%) = OD values (Samples)/OD values (Controls) × 100

**Cell adhesion**

PEEK microcarriers were sterilized by autoclaving and ultraviolet before seeding cells. Then the samples were washed with PBS and soaked with high-glucose Dulbecco’s modified Eagle’s medium (DMEM; Gibco, Invitrogen) supplemented with 10% fetal bovine serum (Zhejiang Tianhang Biotechnology Co., Ltd, China) for night. MC3T3-E1 cells were seeded on each sample in 48-well tissue culture plates at a density of 2 × 10^4^ cells per well followed by culturing for 1,3 and 7 days. Afterwards, the cells were stained with Calcein AM and the nucleus were stained with DAPI using fluorescence microscopy (TE2000U, Nikon, Japan).

**Cell proliferation**

Cell Counting Kit (CCK-8) assay was employed to quantitatively determine the viable MC3T3-E1 cells on the samples. MC3T3-E1 cells were seeded on each sample in the 48-well tissue culture plates at a density of 2 × 10^4^ cells/well and cultured for 1, 3 and 7 days. At every prescribed time point, 30 µL/well CCK-8 was added to the medium. After 2 h of incubation, 200 µL of the medium was transferred to a 96-well plate for measurement. The absorbance was determined at 450 nm using a multifunctional micro-plate scanner (Tecan Infinite M200).

**Decellularization and ECM morphology**

We removed cellular components according to previous protocols.[[2](#_ENREF_2)] The topological PEEK microcarriers after culturing 7 days were placed into the ﬁrst decellularization solution, which contained 1 M NaCl, 10 mM Tris, and 5 mM EDTA (Sigma). The samples were shaken for 1 h at room temperature and rinsed thoroughly with PBS. Then they were placed in a second decellularization solution containing 0.5 wt% SDS, 10 mM Tris, and 25 mM EDTA (Sigma), and shaken for 0.5 h at room temperature. After a PBS wash, the samples were ﬁxed with 4% paraformaldehyde, washed with PBS, and then dehydrated through a graded series of ethanol. Finally the samples were dried and viewed using a scanning electron microscope (SEM, Zeiss, Merlin, Gemini 2, Germany) and Energy dispersive X-ray spectrometry (EDX) (XL-30W/TMP, Philips, Japan).

**Recellularization and cell proliferation**

The ECM encapsulated PEEK microcarriers were sterilized by autoclaving under 121 °C for 20 min and ultraviolet before recellularization. Then the samples were washed with PBS and soaked with culture medium for night. MC3T3-E1 cells were reseeded on each sample in 48-well tissue culture plates at a density of 2 × 10^4^ cells per well followed by culturing for 7 days. Afterwards, the cells were stained with Calcein AM using fluorescence microscopy (TE2000U, Nikon, Japan). Cell Counting Kit (CCK-8) assay was employed to quantitatively determine the viable MC3T3-E1 cells on the samples. At every prescribed time point, 30 µL/well CCK-8 was added to the medium. After 2 h of incubation, 200 µL of the medium was transferred to a 96-well plate for measurement. The absorbance was determined at 450 nm using a multifunctional micro-plate scanner (Tecan Infinite M200).

**References**

1. Xu Y, Han J, Chai Y *et al.* Development of porous chitosan/tripolyphosphate scaffolds with tunable uncross-linking primary amine content for bone tissue engineering. *Materials Science & Engineering C* 2018;85:S0928493117322907.

2. Xing Q, Yates K, Tahtinen M *et al.* Decellularization of Fibroblast Cell Sheets for Natural Extracellular Matrix Scaffold Preparation. *Tissue Eng Part C Methods* 2015;21:77.
