# Supplementary material for: Biomimetic polyetheretherketone microcarriers with specific surface topography and self-secreted extracellular matrix for large-scale cell expansion
Source: Regen Biomater. 2019 Sep 30;7(1):109–18. doi: 10.1093/rb/rbz032 (PMC7233611; doi:10.1093/rb/rbz032)
Supplement: rbz032-Supplementary_data [file rbz032-supplementary_data.zip › rbz032-suppl_data/Responses__to__Reviewers---20190820.docx]

**NOTE:** The revised contents in the manuscript were labeled in RED. Our responses were written in BLUE just after each comment (BLACK) of the reviewers. The revised content of manuscript was briefly shown following our response.

First of all, we would like to express our great appreciation to the reviewers for their helpful comments and suggestions.

**Responses to reviewers’ comments**

**Reviewer: 1**

Comments:

The authors developed an innovative strategy to prepare biomimetic polyetheretherketone microcarriers with specific surface topography and self-secreted ECM. Then they investigated cell adhesion and proliferation behavior of the newly developed microcarriers using MC3T3-E1 pre-osteoblasts. The idea is interesting and the work is attractive for developing new microcarriers with micro- and nano-scale surface feature and improving surface properties in tissue engineering and large-scale cell expansion. The authors also give a thoughtful discussion on the topological feature, cell behavior and correlate it well with the biofunctionalization with self-secreted ECM. Therefore I suggest to accept it after minor revision.

**Response:** Thanks for your comments.

1. The full form of abbreviations should be provided whenever it appears for the first time (such as ECM).

**Response:** Thank you for your suggestion. The full form of abbreviations has been provided whenever it appears first. For example, the full form of ECM is [extracellular](javascript:;) [matrix](javascript:;). Therefore, the title of this manuscript has been revised as "Biomimetic polyetheretherketone microcarriers with specific surface topography and self-secreted [extracellular](javascript:;) [matrix](javascript:;) for large-scale cell expansion".

2) In the abstract, please specify how long the hydrothermal treatment time is to get the resulting microcarriers with size distribution of 350.24±19.44 µm.

**Response:** Thanks for your comments. [Specific](javascript:;) [time](javascript:;) information has been added to get the resulting microcarriers in the abstract.

“After hydrothermal treated for 8 h, the resulting topological PEEK microcarriers exhibit walnut-like surface topography and good sphericity as well as uniform size distribution of 350.24±19.44 µm.”

3) How the authors measure the width between ravine-patterned surface of microcarriers in Figure 1? Please give more detailed description.

**Response:** Thanks for your comments. The measuring method has been added in the Supporting information.

“In Figure 1, we measured the ravine from one side to the other side using Image J software. At least five widths including the widest and narrowest distances were selected for each ravine. The average width between ravine-patterned surface of PEEK microcarriers was shown in Table S1.”

4) The authors should give more detailed parameters of repeating steam sterilization by autoclaving before reseeding cells.

**Response:** Thanks for your comments. The detailed parameters of repeating steam sterilization by autoclaving before reseeding cells have been added in the Supporting information.

“The ECM encapsulated PEEK microcarriers were sterilized by autoclaving under 121 °C for 20 min and ultraviolet before recellularization.”

5) In Supporting information, please give more information about cell culture medium used in cell experiments, such as name, component, manufacturer, and so on.

**Response:** Thanks for your comments. The more information about cell culture medium has been added in the Supporting information.

“**Cell adhesion**

PEEK microcarriers were sterilized by autoclaving and ultraviolet before seeding cells. Then the samples were washed with PBS and soaked with high-glucose Dulbecco’s modified Eagle’s medium (DMEM; Gibco, Invitrogen) supplemented with 10% fetal bovine serum (Zhejiang Tianhang Biotechnology Co., Ltd, China) for night.”

Additionally, some words, sentences and spelling mistakes were corrected. All of these revisions were labeled in Red in the revised manuscript.
